# Supplementary material for: Reprograming skin fibroblasts into Sertoli cells: a patient-specific tool to understand effects of genetic variants on gonadal development
Source: Biol Sex Differ. 2024 Mar 22;15:24. doi: 10.1186/s13293-024-00599-y (PMC10958866; doi:10.1186/s13293-024-00599-y)
Supplement: Supplementary file 1 — Additional file 1: Fig. S1. Standardization of trans-differentiation protocol. (A) Outline of the initial 16 culture conditions designed to attempt fibroblast-Sertoli cell like trans-differentiations. (B) The outcome of these conditions was evaluated by looking for Sertoli marker expression in each of the 16 conditions by qPCR for: SOX9, BMP4, PTGDS and AR. N = 2, n = 6 for each of the qPCRs, error bars represent SEM. (C) Representative images of 1-month live cultures for all these conditions. Missing images either had too low green fluorescence or extensive cell death. Figure S2. 46,XY SLC characterization. Heatmaps showing expression of co-DEGs for 8TF, GFPC, aSC (A) or 6TF, GFPC, aSC (B). Biological process gene ontology (GO) analyses for the co-DEGs of 8TF (C, red) and 6TF (D, blue). (E) RNAseq analysis showing linear scale fold change in expression of indicated markers in 46,XY derived 8TF (red) and 6TF (blue) SLCs over GFPC N = 3, * represent adjusted p value of fold change calculations, ns not shown. (F) Linear scale fold change for indicated markers as determined by qPCR for 8TF (red) and 6TF (blue). The values represent mean ± SEM of the following number of samples (N = biological replicate, n = technical replicate): For 8 TF, PTGDS: N = 9, n = 18; AR: N = 9, n = 12; FSHR: N = 4, n = 16; SOX9: N = 12, n = 25; BMP4: N = 9, n = 18; ACTA2: N = 3, n = 3; DDX4: N = 3, n = 3; FOXL2: N = 3, n = 6. For 6 TF: PTGDS: N = 6, n = 12; AR: N = 6, n = 6; FSHR: N = 9, n = 15; SOX9: N = 6, n = 12; BMP4: N = 6, n = 12; ACTA2: N = 3, n = 3; DDX4: N = 3, n = 3; FOXL2: N = 3, n = 3. * represent p values calculated from unpaired t test conducted between each 8TF or 6TF and GFPC. Figure S3. Morphometry of 46,XY DSD and 46,XX SLC. Representative images of GFPC and 8TF SLCs on Day7 and Day28; Shape factor and area quantifications for GFPC, 8TF and 6TF SLCs (N = 3, n = 50–60 for each group). * represent p values calculated from Mann–Whitney statistical tests conducted between the in [file 13293_2024_599_MOESM1_ESM.docx]

**Additional Figs:**
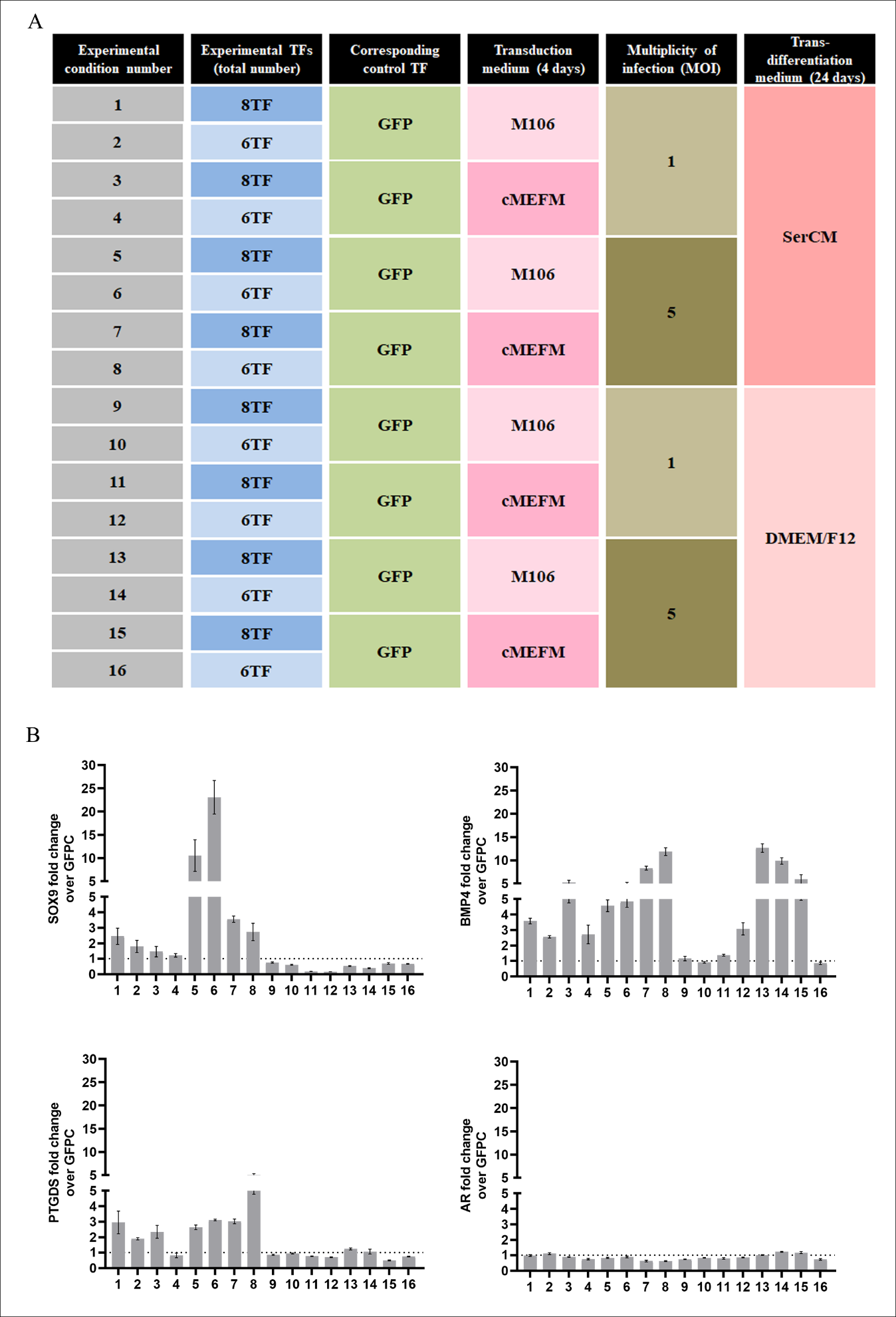


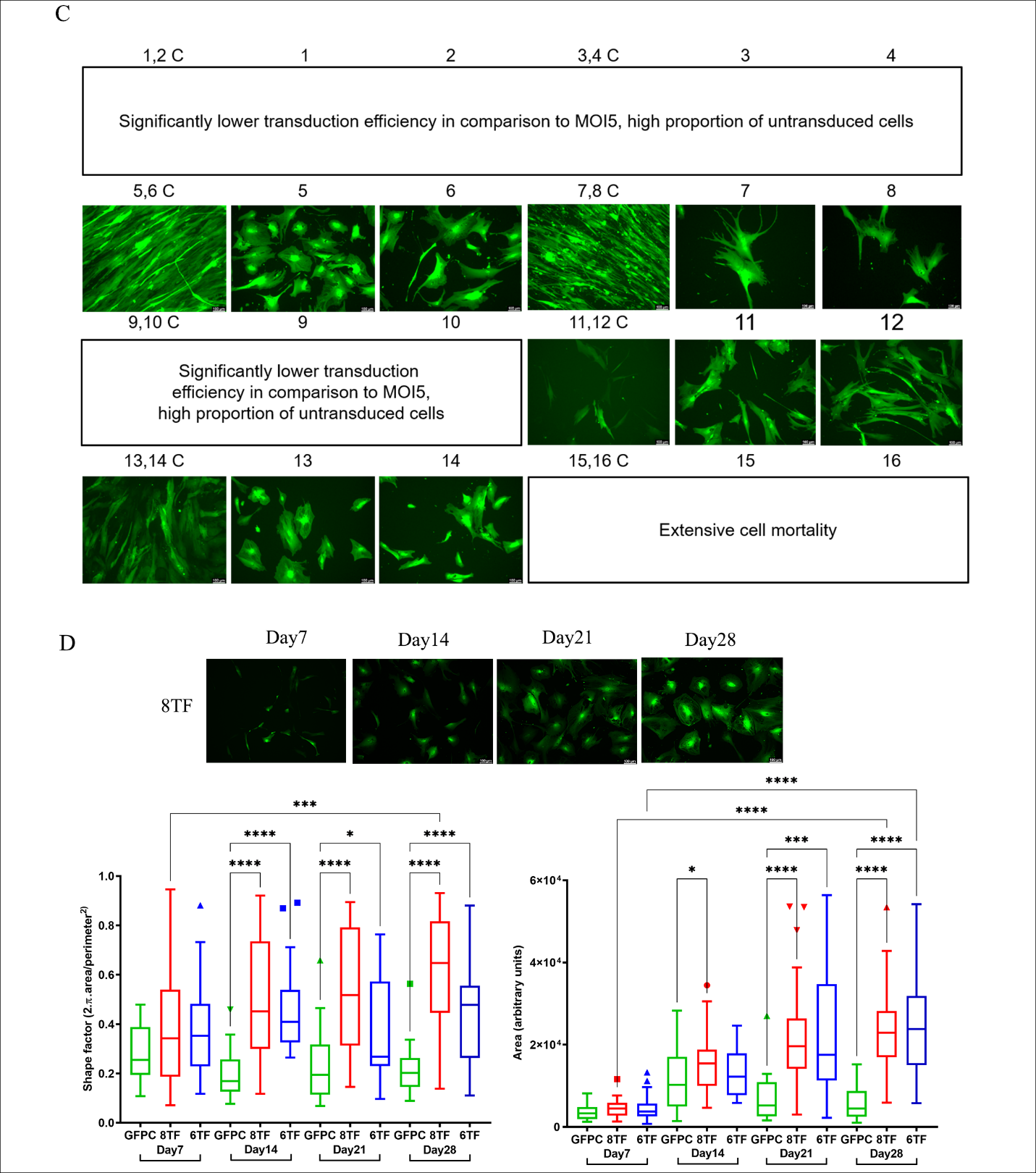


**Fig. S1: Standardization of trans-differentiation protocol**. (A) Outline of the initial 16 culture conditions designed to attempt fibroblast-Sertoli cell like trans-differentiations. (B) The outcome of these conditions was evaluated by looking for Sertoli marker expression in each of the 16 conditions by qPCR for: *SOX9*, *BMP4*, *PTGDS* and *AR*. N=2, n=6 for each of the qPCRs, error bars represent SEM. (C) Representative images of 1-month live cultures for all these conditions. Missing images either had too low green fluorescence or extensive cell death.


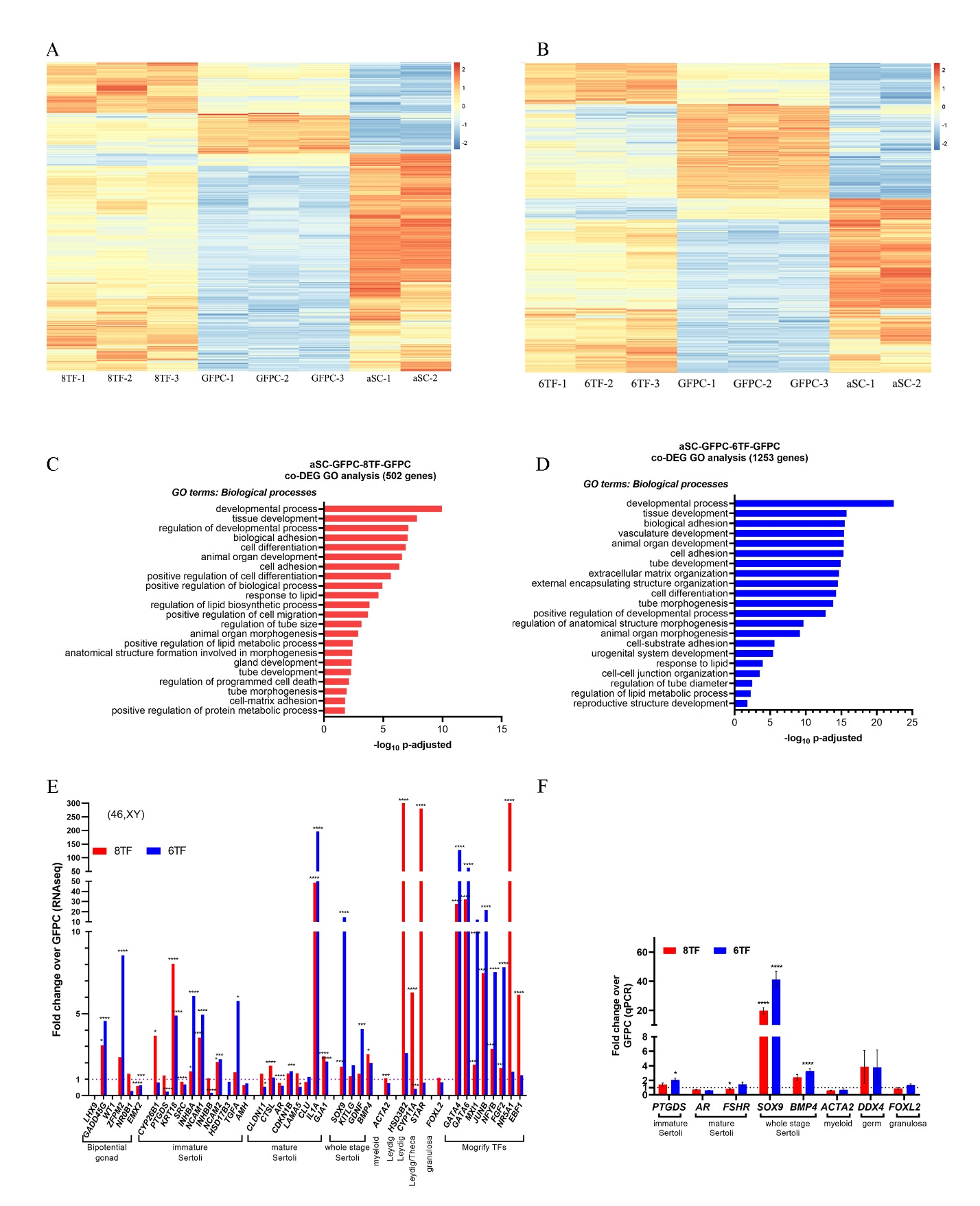


**Fig. S2: 46,XY SLC characterization.**  Heatmaps showing expression of co-DEGs for 8TF, GFPC, aSC (A) or 6TF, GFPC, aSC (B). Biological process gene ontology (GO) analyses for the co-DEGs of 8TF (C, red) and 6TF (D, blue). (E) RNAseq analysis showing linear scale fold change in expression of indicated markers in 46,XY derived 8TF (red) and 6TF (blue) SLCs over GFPC N=3, * represent adjusted p value of fold change calculations, ns not shown. (F) Linear scale fold change for indicated markers as determined by qPCR for 8TF (red) and 6TF (blue). The values represent mean +/- SEM of the following number of samples (N=biological replicate, n=technical replicate): For 8 TF, *PTGDS*: N=9, n=18; *AR*: N=9, n=12; *FSHR*: N=4, n=16; *SOX9*: N=12, n=25; *BMP4*: N=9, n=18; *ACTA2*: N=3, n=3; *DDX4*: N=3, n=3; *FOXL2*: N=3, n=6. For 6 TF: *PTGDS*: N=6, n=12; *AR*: N=6, n= 6; *FSHR*: N=9, n=15; *SOX9*: N=6, n=12; *BMP4*: N=6, n=12; *ACTA2*: N=3, n=3; *DDX4*: N=3, n=3; *FOXL2*: N=3, n=3. * represent p values calculated from unpaired t test conducted between each 8TF or 6TF and GFPC.


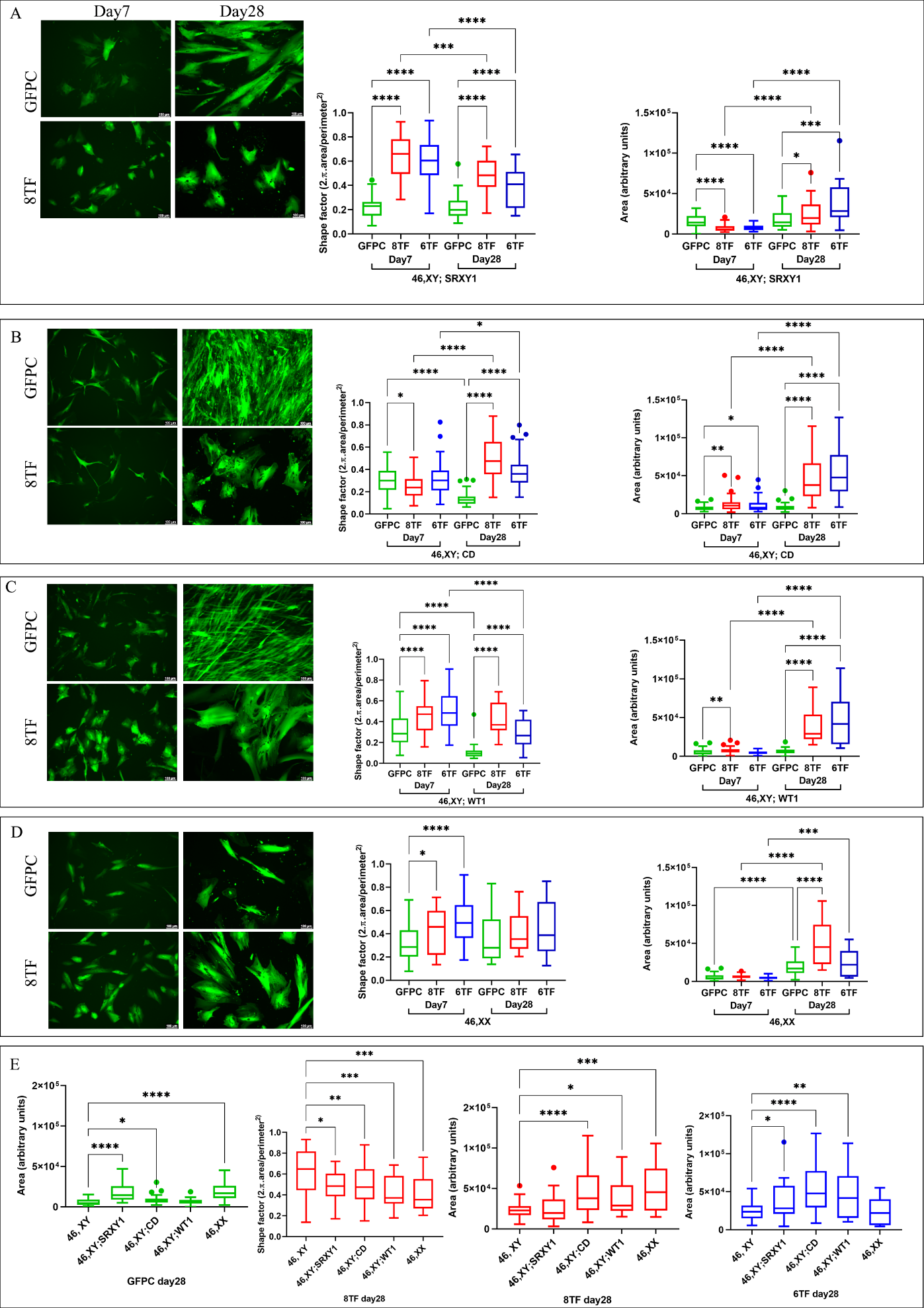


**Fig. S3: Morphometry of 46,XY DSD and 46,XX SLC.** Representative images of GFPC and 8TF SLCs on Day7 and Day28; Shape factor and area quantifications for GFPC, 8TF and 6TF SLCs (N=3, n=50-60 for each group). * represent p values calculated from Mann-Whitney statistical tests conducted between the indicated groups for (A). 46, XY; SRXY1 (B) 46,XY; CD; (C) 46,XY; WT1 and (D) 46,XX; non significant p value comparisons are omitted. (E) GFPC area factor, 8TF shape factor, 8TF area factor, 6TF area factor quantifications comparing 46, XY DSD and 46,XX genetic backgrounds with 46, XY.


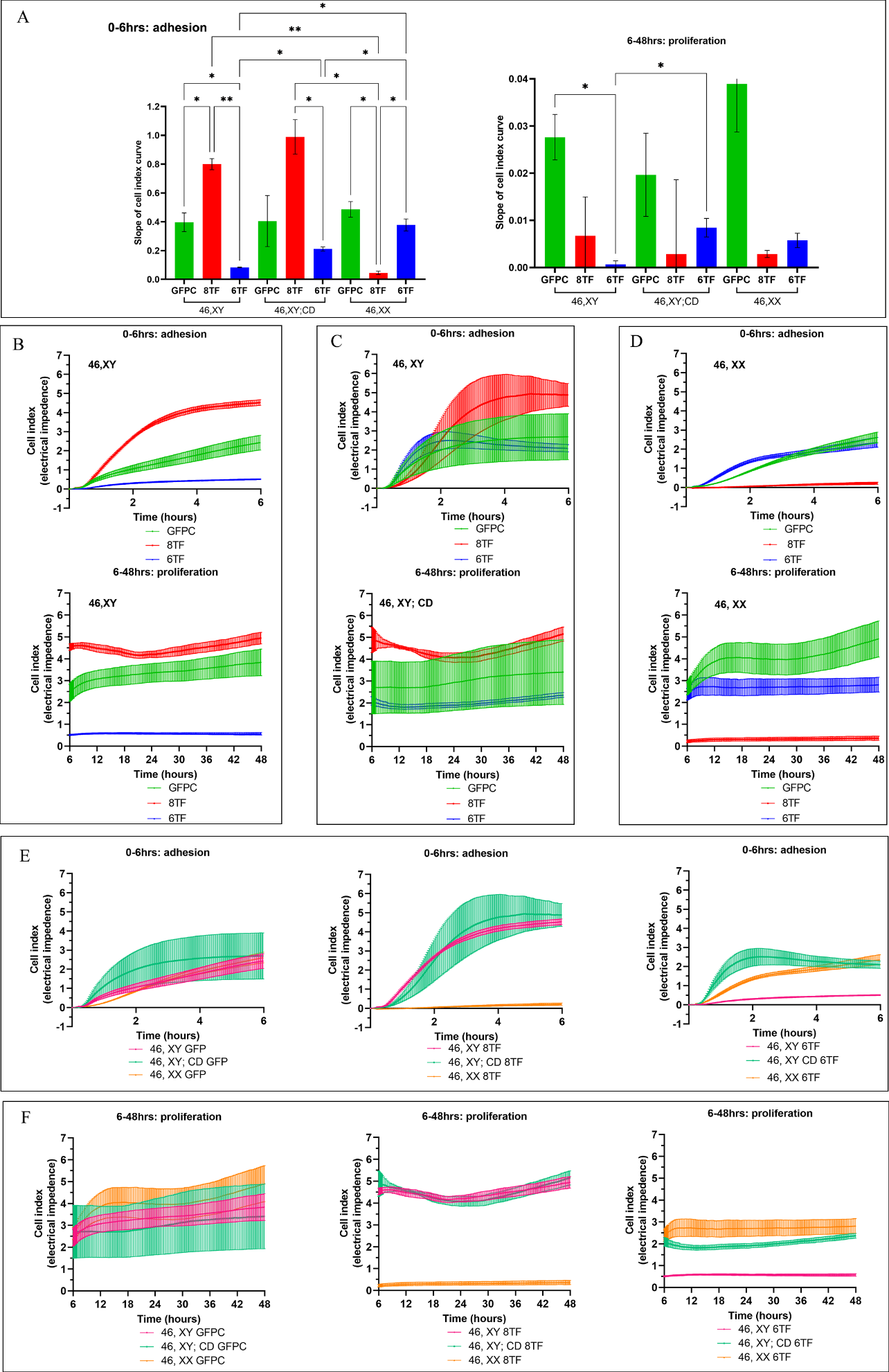


**Fig. S4: Cell index (CI) curves of xCELLigence assays.** (A) Slope of 0-6 hours cell index (CI) adhesion and 6-48 hrs proliferation curves for 1-month-old GFPC, 8TF and 6TF SLC derived from fibroblasts of indicated genetic backgrounds. All the experimental readings represent an average of three biological replicates (N=3), * represent p values calculated from one way ANOVA test conducted amongst the three groups within each graph, ns is not shown. 0-6 hours CI adhesion curves and 6-48 hrs CI proliferation curves for 1-month-old GFPC, 8TF SLC and 6TF SLC derived from 46,XY (B), 46,XY;CD (C) and 46, XX (D). (E): Alternative representation of 0-6 hours cell index (CI) adhesion curves and (F) 6-48 hrs CI proliferation curves for 46,XY, 46,XY with campomelic dysplasia (CD), or 46,XX-derived GFPC, 8TF SLCs and 6TF SLCs. All the data points represent an average plus SEM of three biological replicates (N=3).


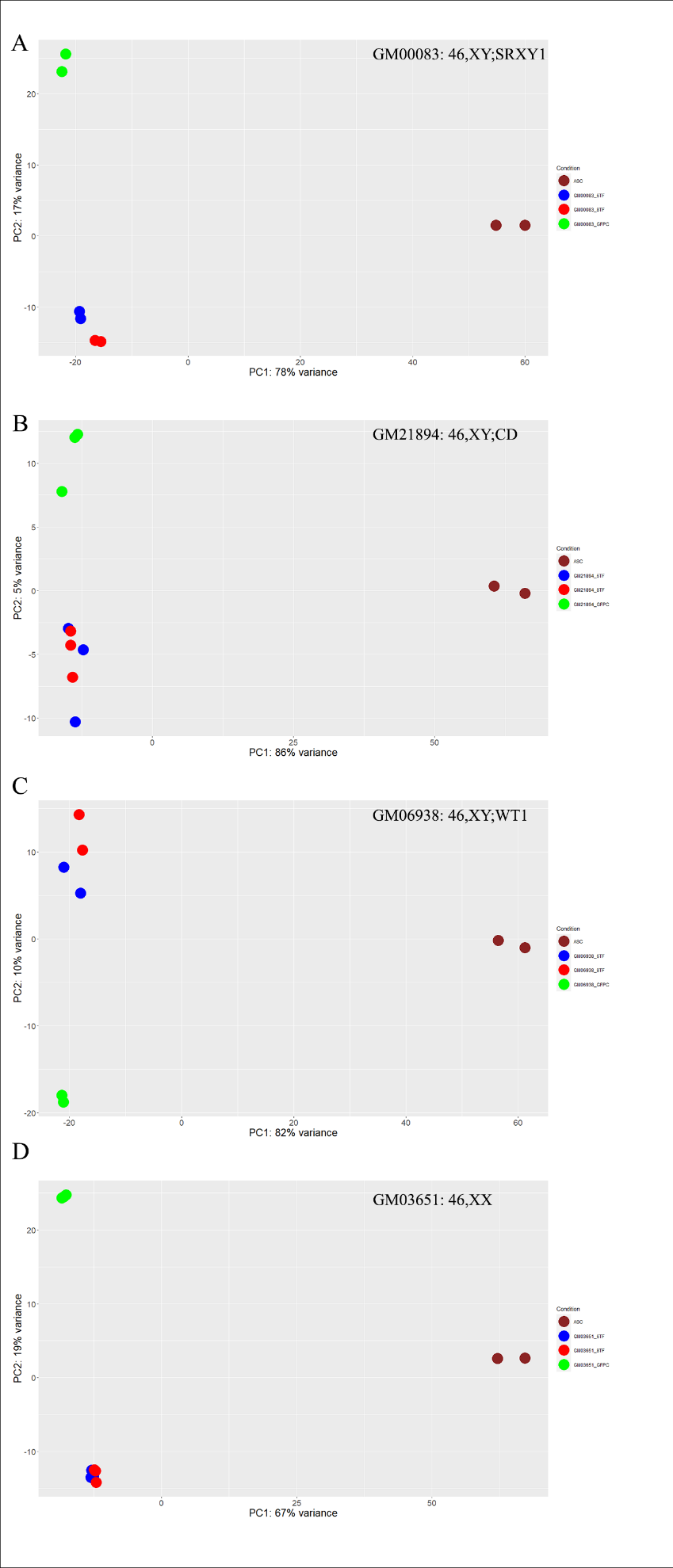


**Fig. S5-1: Principal Component Analysis of RNAseq** transcriptomes comparing all the replicate samples for aSC (in brown), GFPC (green), 8TF (red), 6TF (blue) derived from (A) GM00083: 46,XY; SRXY1, (B) GM21894: 46,XY; CD, (C) GM06938: 46,XY; WT1 and (D) GM03651: 46,XX.


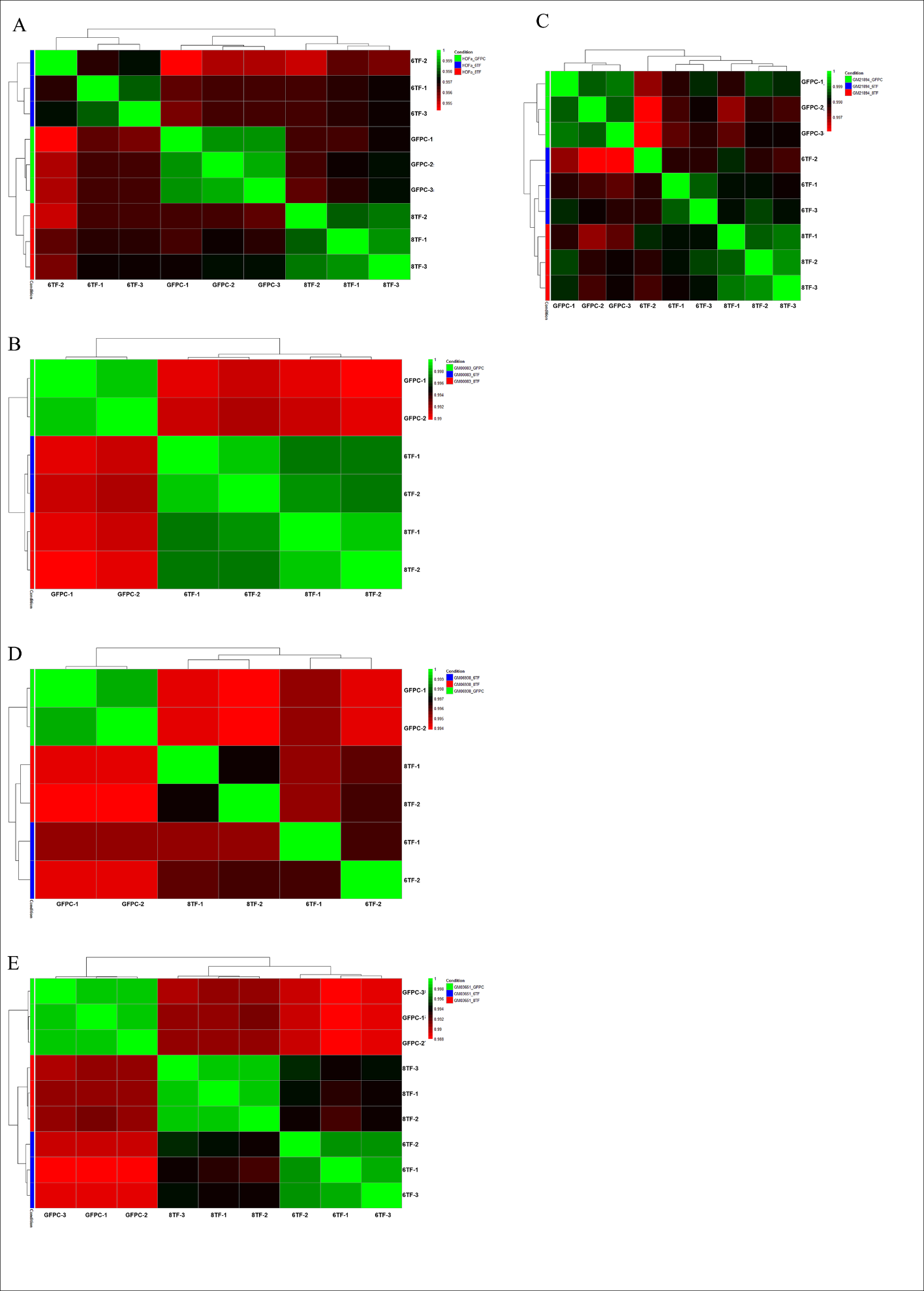


**Fig. S5-2: RNASeq sample replicates cluster together.** Correlation plots for each of the 8TF (red bars along Y axes), 6TF (blue bars) and GFPC (green bars) RNA seq samples of (A) 46,XY, (B) 46,XY; SRXY1, (C) 46,XY; CD ,(D) 46,XY;WT1 and (E) 46,XX.


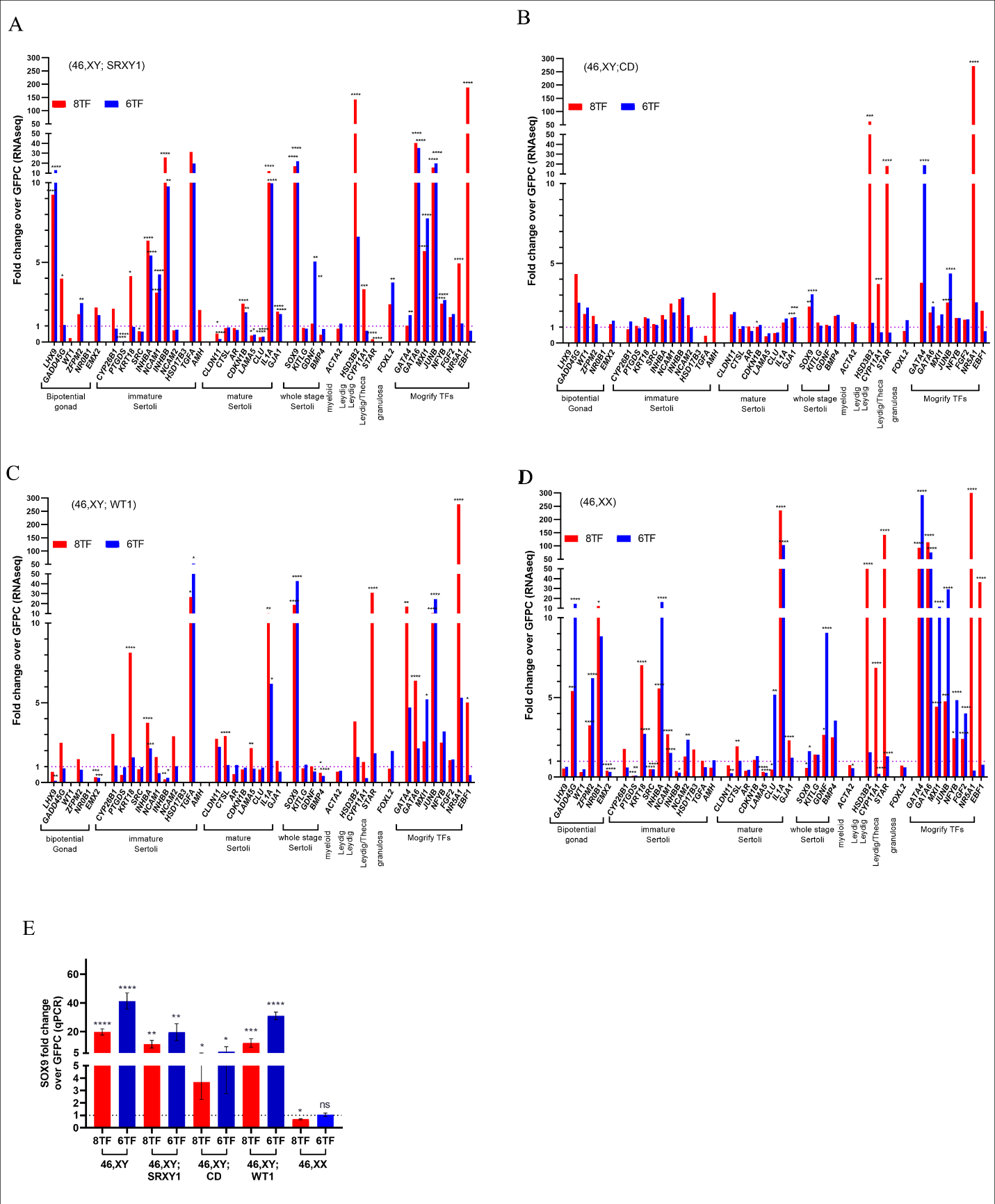


**Fig. S5-3: A wide range of gonadal markers and transduced Mogrify TFs show expression in SLC samples.** RNAseq analysis shows linear scale fold change in expression of indicated markers in 8TF- (red) or 6TF -(blue) derived SLCs in comparison to GFPC for (A) 46, XY; SRXY1 (B) 46,XY;CD (C) 46,XY;WT1 and (D) 46,XX. N=3, * represent adjusted p value of fold change calculations between experimental and respective control, ns not shown. (E): Fold change for *SOX9* as determined by qPCR, in both 8TF (red) or 6TF (blue) SLC over respective GFPC for the indicated cell lines. N=5, n=25 for 46,XY, N=3,n=3 each for 46,XY;SRXY1, 46,XY;CD, 46,CD;WT1 and 46,XX, error bars represent SEM. * represent p values calculated from unpaired t test conducted between each experimental and its corresponding GFPC, ns not shown.


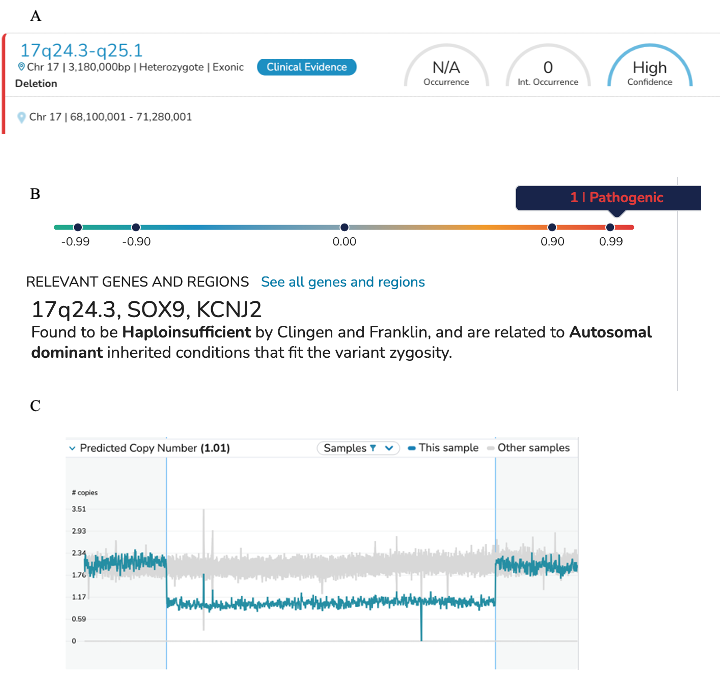


**Fig. S5-4: Identification of the *SOX9* variant in the 46,XY CD cell line.**

DNA extracted from the Coriell cell lines GM03368 (46, XY; SRXY1 of unknown cause) and GM21894 (46,XY; CD) was subjected to exome sequencing, using the Agilent SureSelect v.6 capture kit. Sequencing was performed at Novogene. Average depth of coverage was 174x and 189x, respectively. Exome did not identify a causative variant in either sample. GM21894 (46,XY; CD) was then subjected to PCR-free whole-genome sequencing (performed at the Broad Institute, Cambridge, MA), with a resulting average read depth of 45x. Sequence analysis was performed on the Franklin (Genoox, Inc.) platform, using both the single nucleotide variants and structural variants pipelines (A). This identified a 3.2 Mb heterozygous deletion on chromosome 17q24.3-25.1 (B), including the entire *SOX9* gene (and 44 other genes), at chromosomal coordinates chr17:68,100,001-71,280,001 (on the hg19 human genome reference) (C).

**Table S1. List of marker genes**

| **Symbol** | **Description** | **Cell type** | **Stage** | **Species** | **References** |
| --- | --- | --- | --- | --- | --- |
| *CYP26B1* | Cytochrome P450 Family 26 Subfamily B Member 1 | Sertoli | immature | Mouse, Human | [56–58] |
| *PTGDS* | Prostaglandin D2 Synthase | Sertoli | immature | Mouse, Human | [59–62] |
| *KRT18* | Keratin 18 | Sertoli | immature | Mouse, Human | [63–68] |
| *SRC* | SRC Proto-Oncogene, Non-Receptor Tyrosine Kinase | Sertoli | immature | Mouse, Rat, Human | [34, 69–71] |
| *INHBA* | Inhibin Subunit Beta A | Sertoli | immature | Mouse, Rat, Human | [72, 73] |
| *INHBB* | Inhibin Subunit Beta B | Sertoli | immature | Mouse, Rat, Human | [72, 73] |
| *NCAM1* | Neural Cell Adhesion Molecule 1 | Sertoli | immature | Rat, Mouse | [74–78] |
| *NCAM2* | Neural Cell Adhesion Molecule 2 | Sertoli | immature | Rat, Mouse | [74–78] |
| *HSD17B3* | Hydroxysteroid 17-Beta Dehydrogenase 3 | Sertoli | immature | Mouse, Human | [62, 79] |
| *TGFA* | Transforming Growth Factor Alpha | Sertoli | immature | Mouse, Rat, Human | [51, 52, 80] |
| *CLDN11* | Claudin 11 | Sertoli | mature | Mouse, Rat, Human | [81–83] |
| *CTSL* | Cathepsin L | Sertoli | mature | Mouse, Rat, Human | [84, 85] |
| *AR* | Androgen receptor | Sertoli | mature | Human | [86] |
| *CDKN1B/* *p27^Kip1^* | Cyclin Dependent Kinase Inhibitor 1B | Sertoli | mature | Mouse, Human | [87, 88] |
| *LAMA5* | Laminin Subunit Alpha 5 | Sertoli | mature | Rat, Human | [89–92] |
| *CLU* | Clusterin | Sertoli | mature | Mouse, Rat, Human | [93–95] |
| *IL1A* | Interleukin 1 Alpha | Sertoli | mature | Mouse, Rat, Human | [96–100] |
| *CX43/GJA1* | Connexin-43/ Gap Junction Protein Alpha 1 | Sertoli | mature | Mouse, Rat, Human | [96, 101, 102] |
| *FSHR* | Follicle Stimulating Hormone Receptor | Sertoli | mature | Mouse, Rat, Human | [103–105] |
| *SOX9* | SRY-Box Transcription Factor 9 | Sertoli | all-stage | Mouse, Rat, Human | [106–110] |
| *KITLG/SCF* | KIT Ligand/Stem cell factor | Sertoli | all-stage | Mouse, Rat, Human | [27, 75, 111] |
| *GDNF* | Glial Cell Derived Neurotrophic Factor | Sertoli | all-stage | Mouse, Human | [76, 112–115] |
| *BMP4* | Bone Morphogenetic Protein 4 | Sertoli | all-stage | Mouse, Human | [116–118] |
| *DMRT1* | Doublesex And Mab-3 Related Transcription Factor 1 | Sertoli | immature | Mouse, Human | [119–123] |
| *AMH* | Anti-Mullerian Hormone | Sertoli | immature | Human | [72, 124] |
| *DHH* | Desert Hedgehog Signaling Molecule | Sertoli | all-stage | Mouse | [125–127] |
| *ACTA2/SMA* | Actin Alpha 2, Smooth Muscle/Smooth muscle actin | Peritubular myoid | - | Mouse, Human | [128] |
| *HSD3B2* | Hydroxy-Delta-5-Steroid Dehydrogenase, 3 Beta- And Steroid Delta-Isomerase 2 | Leydig | - | Human | [129, 130] |
| *CYP11A* | Cytochrome P450 Family 11 Subfamily A Member 1 | Leydig | - | Mouse, Human | [131–133] |
| *DDX4/VASA* | DEAD-Box Helicase 4 | germ cell | - | Vertebrates,  invertebrates | [134–136] |
| *FOXL2* | Forkhead Box L2 | granulosa | - | Mouse, human | [137–139] |

56. Kumar S, Chatzi C, Brade T, Cunningham TJ, Zhao X, Duester G.

Sex-specific timing of meiotic initiation is regulated by Cyp26b1

independent of retinoic acid signalling. Nat Commun. 2011;2(1):1–8.

57. Childs AJ, Cowan G, Kinnell HL, Anderson RA, Saunders PTK. Retinoic

acid signalling and the control of meiotic entry in the human fetal

gonad. PLoS ONE. 2011;6(6): e20249.

58. Mcclelland K, Bowles J, Koopman P. Male sex determination: insights

into molecular mechanisms. Asian J Androl. 2012;14:164–71.

59. Loveland KL, Zlatic K, Stein-Oakley A, Risbridger G, deKretser DM.

Platelet-derived growth factor ligand and receptor subunit mRNA

in the Sertoli and Leydig cells of the rat testis. Mol Cell Endocrinol.

1995;108(1–2):155–9.

60. Malki S, Nef S, Notarnicola C, Thevenet L, Gasca S, M.jean C,

et al. Prostaglandin D2 induces nuclear import of the sex-determining

factor SOX9 via its cAMP-PKA phosphorylation. EMBO J.

2005;24(10):1798–809.

61. Moniot B, Declosmenil F, Barrionuevo F, Scherer G, Aritake K, Malki

S, et al. The PGD2 pathway, independently of FGF9, amplifies SOX9

activity in Sertoli cells during male sexual differentiation. Development.

2009;136(11):1813–21.

62. St.vant I, Neirijnck Y, Borel C, Escoffier J, Smith LB, Antonarakis SE, et al.

Deciphering cell lineage specification during male sex determination

with single-cell RNA sequencing. Cell Rep. 2018;22(6):1589–99.

63. Kruse R, Eigelshoven S, Kaiser A, Ruzicka T, Neumann NJ. Cytokeratin

18 expression in immature Sertoli cells: co-localization with interstitial

lymphocytic infiltrates. Folia Histochem Cytobiol. 2009;47(1):127–30.

64. Franke FE, Pauls K, Rey R, Marks A, Bergmann M, Steger K. Differentiation

markers of Sertoli cells and germ cells in fetal and early postnatal

human testis. Anat Embryol (Berl). 2004;209(2):169–77.

65. Nicholls PK, Stanton PG, Chen JL, Olcorn JS, Haverfield JT, Qian H, et al.

Activin signaling regulates sertoli cell differentiation and function.

Endocrinology. 2012;153(12):6065–77.

66. Steger K, Rey R, Louis F, Kliesch S, Behre HM, Nieschlag E, et al. Reversion

of the differentiated phenotype and maturation block in Sertoli cells in

pathological human. Hum Reprod. 1999;14(1):136–43.

67. Steger K, Rey R, Kliesch S, Louis F, Schleicher G, Bergmann M.

Immunohistochemical detection of immature Sertoli cell markers in

testicular tissue of infertile adult men: a preliminary study. Int J Androl.

1996;19(2):122–8.

68. Stosiek P, Kasper M, Karsten U. Expression of cytokeratins 8 and 18 in

human Sertoli cells of immature and atrophic seminiferous tubules.

Differentiation. 1990;43(1):66–70.

69. Xiao X, Mruk DD, Cheng FL, Cheng CY. C-Src and c-Yes are two unlikely

partners of spermatogenesis and their roles in blood-testis barrier

dynamics. Adv Exp Med Biol. 2013;763:295–317.

70. Lee NPY, Cheng CY. Protein kinases and adherens junction dynamics

in the seminiferous epithelium of the rat testis. J Cell Physiol.

2005;202(2):344–60.

71. Xiao X, Mruk DD, Wong EWP, Lee WM, Han D, Wong CKC, et al. Differential

effects of c-Src and c-Yes on the endocytic vesicle-mediated

trafficking events at the Sertoli cell blood-testis barrier: an in vitro study.

Am J Physiol Endocrinol Metab. 2014;307(7):E553–62.

72. Hero M, Tommiska J, Vaaralahti K, Laitinen EM, Sipil. I, Puhakka L, et al.

Circulating anti Mullerian hormone levels in boys decline during early

puberty and correlate with inhibin B. Fertil Steril. 2012;97(5):1242–7.

73. Majdic G, McNeilly AS, Sharpe RM, Evans LR, Groome NP, Saunders PTK.

Testicular expression of inhibin and activin subunits and follistatin in

the rat and human fetus and neonate and during postnatal development

in the rat. Endocrinology. 1997;138(5):2136–47.

74. Wang Q, Liu H, Shi Y, Pan Z, Wang J. Activation of the GFRa1/NCAM

pathway stimulates Sertoli cell proliferation in vitro. Belg J Zool.

2008;138.

75. Wang H, Wen L, Yuan Q, Sun M, Niu M, He Z. Establishment and

applications of male germ cell and Sertoli cell lines. Reproduction.

2016;152(2):R31-40.

76. Yang Y, Han C. GDNF stimulates the proliferation of cultured mouse

immature Sertoli cells via its receptor subunit NCAM and ERK1/2 signaling

pathway. BMC Cell Biol. 2010;11(1):1–10.

77. Orth JM, Jester WF. NCAM mediates adhesion between gonocytes

and sertoli cells in cocultures from testes of neonatal rats. J Androl.

1995;16(5):389–99.

78. Laslett AL, Li LH, Jester WF, Orth JM. Thyroid hormone down-regulates

neural cell adhesion molecule expression and affects attachment

of gonocytes in sertoli cell-gonocyte cocultures. Endocrinology.

2000;141(5):1633–41.

79. Hakkarainen J, Zhang FP, Jokela H, Mayerhofer A, Behr R, Cisneros-

Montalvo S, et al. Hydroxysteroid (17β) dehydrogenase 1 expressed by

Sertoli cells contributes to steroid synthesis and is required for male

fertility. FASEB J. 2018;32(6):3229–41.

80. Petersen C, Boitani C, Fr.ysa B, S.der O. Transforming growth factor-α

stimulates proliferation of rat sertoli cells. Mol Cell Endocrinol.

2001;181(1–2):221–7.

81. McCabe M, Foo C, Dinger M, Smooker P, Stanton P. Claudin-11 and

occludin are major contributors to Sertoli cell tight junction function,

in vitro. Asian J Androl. 2016;18(4):620–6.

82. Stammler A, Lüftner BU, Kliesch S, Weidner W, Bergmann M, Middendorff

R, et al. Highly conserved testicular localization of claudin-11 in

normal and impaired spermatogenesis. PLoS One. 2016;11(8).

83. Hellani A, Ji J, Mauduit C, Deschildre C, Tabone E, Benahmed M.

Developmental and hormonal regulation of the expression of

oligodendrocyte-specific protein/claudin 11 in mouse testis. Endocrinology.

2000;141(8):3012–9.

84. Gye MC, Kim ST. Expression of cathepsin L in human testis under

diverse infertility conditions. Arch Androl. 2004;50(3):187–91.

85. Charron M, Chern JY, Wright WW. The cathepsin L first intron stimulates

gene expression in rat Sertoli cells. Biol Reprod. 2007;76(5):813–24.

86. Su.rez-Quian CA, Mart.nez-Garc.a F, Nistal M, Regadera J. Androgen

receptor distribution in adult human testis1. J Clin Endocrinol Metab.

1999;84(1):350–8.

87. Holsberger DR, Buchold GM, Leal MC, Kiesewetter SE, O’Brien DA, Hess

RA, et al. Cell-cycle inhibitors p27Kip1 and p21cip1 regulate murine

sertoli cell proliferation. Biol Reprod. 2005;72(6):1429–36.

88. Beumer TL, Kiyokawa H, Roepers-Gajadien HL, Van Den Bos LAC, Lock

TMTW, Gademan IS, et al. Regulatory role of p27 kip1 in the mouse and

human testis*. 1999.

89. Pelliniemi LJ, Fr.jdman K. Structural and regulatory macromolecules in

sex differentiation of gonads. J Exp Zool. 2001; 523–8.

90. Richardson LL, Kleinman HK, Dym M. Basement membrane gene

expression by Sertoli and peritubular myoid cells in vitro in the rat. Biol

Reprod. 1995;52(2):320–30.

91. Ulisse S, Rucci N, Piersanti D, Carosa E, Graziano FM, Pavan A, et al.

Regulation by thyroid hormone of the expression of basement

membrane components in rat prepubertal Sertoli cells. Endocrinology.

1998;139(2):741–7.

92. Virtanen I, Lohi J, Tani T, Korhonen M, Burgeson RE, Lehto VP, et al.

Distinct changes in the laminin composition of basement membranes

in human seminiferous tubules during development and degeneration.

Am J Pathol. 1997;150(4):1421–31.

93. Sato T, Katagiri K, Gohbara A, Inoue K, Ogonuki N, Ogura A, et al. In vitro

production of functional sperm in cultured neonatal mouse testes.

Nature. 2011;471(7339):504–8.

94. Grima J, Pineau C, Bardin CW, Cheng CY. Rat Sertoli cell clusterin,

α2-macroglobulin, and testins: Biosynthesis and differential regulation

by germ cells. Mol Cell Endocrinol. 1992;89(1–2):127–40.

95. O’Bryan MK, Mallidis C, Murphy BF, Baker HWG. Immunohistological

localization of clusterin in the male genital tract in humans and marmosets.

Biol Reprod. 1994;50(3):502–9.

96. St.phan JP, Syed V, J.gou B. Regulation of sertoli cell IL-1 and IL-6

production in vitro. Mol Cell Endocrinol. 1997;134(2):109–18.

97. G.rard N, Syed V, Bardin W, Genetet N, J.gou B. Sertoli cells are the

site of interleukin-1α synthesis in rat testis. Mol Cell Endocrinol.

1991;82(1):R13–6.

98. Cudicini C, Lejeune H, Gomez E, Bosmans E, Ballet F, Saez J, et al. Human

leydig cells and sertoli cells are producers of interleukins-1 and-6. J Clin

Endocrinol Metab. 1997;82(5):1426–33.

99. S.der O, Syed V, Callard GV, Toppari J, P.ll.nen P, Parvinen M, et al. Production

and secretion of an interleukin-l-like factor is stage-dependent

and correlates with spermatogonial DNA synthesis in the rat seminiferous

epithelium. Int J Androl. 1991;14(3):223–31.

100. Jonsson CK, Zetterstrӧm RH, Zetterstrӧm Z, Holst M, Parvinen M, Sӧder

O, et al. Constitutive expression of interleukin-1 messenger ribonucleic

acid in rat sertoli cells is dependent upon interaction with germ cells.

Endocrinology. 1999;140.

101. Hollenbach J, Jung K, Noelke J, Gasse H, Pfarrer C, Koy M, et al. Loss of

connexin43 in murine Sertoli cells and its effect on blood-testis barrier

formation and dynamics. PLoS ONE. 2018;13(6): e0198100.

102. Giese S, Hossain H, Markmann M, Chakraborty T, Tchatalbachev S,

Guillou F, et al. Sertoli-cell-specific knockout of connexin 43 leads to

multiple alterations in testicular gene expression in prepubertal mice.

DMM Dis Models Mech. 2012;5(6):895–913.

103. Heckert LL, Griswold MD. Expression of follicle-stimulating hormone

receptor mRNA in rat testes and sertoli cells. Mol Endocrinol.

1991;5(5):670–7.

104. Zirkin BR, Awoniyi C, Griswold MD, Russell LD, Sharpeh R. Is FSH

required for adult spermatogenesis? J Androl. 1994;15:273–6.

105. B.ckers TM, Nieschlag E, Kreutz MR, Bergmann M. Localization of

follicle-stimulating hormone (FSH) immunoreactivity and hormone

receptor mRNA in testicular tissue of infertile men. Cell Tissue Res.

1994;278(3):595–600.

106. De Santa BP, Bonneaud N, Boizet B, Desclozeaux M, Moniot B, Sudbeck

P, et al. Direct Interaction of SRY-related protein SOX9 and steroidogenic

factor 1 regulates transcription of the human anti-Müllerian hormone

gene. Mol Cell Biol. 1998;18(11):6653–65.

107. Hanley NA, Hagan DM, Clement-Jones M, Ball SG, Strachan T, Salas-

Cort.s L, et al. SRY, SOX9, and DAX1 expression patterns during

human sex determination and gonadal development. Mech Dev.

2000;91(1–2):403–7.

108. Da SSM, Hacker A, Harley V, Goodfellow P, Swain A, Lovell-Badge R. Sox9

expression during gonadal development implies a conserved role for

the gene in testis differentiation in mammals and birds. Nat Genet.

1996;14(1):62–8.

109. Koopman P. Sry, Sox9 and mammalian sex determination. EXS. EXS;

2001. p. 25–56.

110. Kent J, Wheatley SC, Andrews JE, Sinclair AH, Koopman P. A malespecific

role for SOX9 in vertebrate sex determination. Development.

1996;122(9):2813–22.

111. Steinmetz R, Lazzaro N, Rothrock JK, Pescovitz OH. Effects of growth

hormone-releasing hormone-related peptide on stem cell factor

expression in cultured rat Sertoli cells. Endocrine. 2000;12(3):323–7.

112. Meng X, Lindahl M, Hyv.nen ME, Parvinen M, De Rooij DG, Hess MW,

et al. Regulation of cell fate decision of undifferentiated spermatogonia

by GDNF. Science (1979). 2000;287(5457):1489–93.

113. Viglietto G, Dolci S, Bruni P, Baldassarre G, Chiariotti L, Melillo RM, et al.

Glial cell line-derived neutrotrophic factor and neurturin can act as

paracrine growth factors stimulating DNA synthesis of Ret-expressing

spermatogonia. Int J Oncol. 2000;16(4):689–94.

114. Hu J, Shima H, Nakagawa H. Glial cell line-derived neurotropic factor

stimulates sertoli cell proliferation in the early postnatal period of rat

testis development. Endocrinology. 1999;140(8):3416–21.

115. Wu Z, Templeman JL, Smith RA, Mackay S. Effects of glial cell linederived

neurotrophic factor on isolated developing mouse Sertoli cells

in vitro. J Anat. 2005;206(2):175–84.

116. Hai Y, Sun M, Niu M, Yuan Q, Guo Y, Li Z, et al. BMP4 promotes

human Sertoli cell proliferation via Smad1/5 and ID2/3 pathway

and its abnormality is associated with azoospermia. Discov Med.

2015;19(105):311–25.

117. Hu J, Chen YX, Wang D, Qi X, Li TG, Hao J, et al. Developmental expression

and function of Bmp4 in spermatogenesis and in maintaining

epididymal integrity. 2004;

118. Pellegrini M, Grimaldi P, Rossi P, Geremia R, Dolci S. Developmental

expression of BMP4ÄALK3ÄSMAD5 signaling pathway in the mouse

testis: a potential role of BMP4 in spermatogonia differentiation. J Cell

Sci. 2003;116:3363–72.

119. Raymond CS, Murphy MW, O’Sullivan MG, Bardwell VJ, Zarkower D.

Dmrt1, a gene related to worm and fly sexual regulators, is required for

mammalian testis differentiation. Genes Dev. 2000;14(20):2587–95.

120. Lei N, Hornbaker KI, Rice DA, Karpova T, Agbor VA, Heckert LL.

Sex-specific differences in mouse DMRT1 expression are both cell

type- and stage-dependent during gonad development. Biol Reprod.

2007;77(3):466–75.

121. Malolina EA, Kulibin AY. The rete testis harbors Sertoli-like cells capable

of expressing DMRT1. Reproduction. 2019;158(5):399–413.

122. Huang S, Ye L, Chen H. Sex determination and maintenance: the role of

DMRT1 and FOXL2. Asian J Androl. 2017;19:619–24.

123. J.rgensen A, Nielsen JE, Blomberg Jensen M, Gr.m N, Rajpert-De

Meyts E. Analysis of meiosis regulators in human gonads: a sexually

dimorphic spatio-temporal expression pattern suggests involvement of

DMRT1 in meiotic entry. Mol Hum Reprod. 2012;18(11):523–34.

124. Blagosklonova O, Joanne C, Roux C, Bittard H, Fellmann F, Bresson JL.

Absence of anti-Müllerian hormone (AMH) and M2A immunoreactivities

in Sertoli cell-only syndrome and maturation arrest with and

without AZF microdeletions. Hum Reprod. 2002;17(8):2062–5.

125. Beverdam A, Wilhelm D, Koopman P. Molecular characterization of

three gonad cell lines. Cytogenet Genome Res. 2003. 242–9.

126. Bitgood MJ, Shen L, McMahon AP. Sertoli cell signaling by Desert

hedgehog regulates the male germline. Curr Biol. 1996;6(3):298–304.

127. Clark AM, Garland KK, Russell LD. Desert hedgehog (Dhh) gene is

required in the mouse testis for formation of adult-type Leydig cells

and normal development of peritubular cells and seminiferous tubules.

Biol Reprod. 2000;63(6):1825–38.

128. Mayerhofer A. Human testicular peritubular cells: more than meets the

eye. Reproduction. 2013;145(5).

129. Burckhardt MA, Udhane SS, Marti N, Schnyder I, Tapia C, Nielsen JE, et al.

Human 3β-hydroxysteroid dehydrogenase deficiency seems to affect

fertility but may not harbor a tumor risk: lesson from an experiment of

nature. Eur J Endocrinol. 2015;173(5):K1-12.

130. Flück CE, Pandey AV. Steroidogenesis of the testis-new genes and pathways

St.ro.dogen.se testiculaire-nouveaux g.nes et nouvelles voies.

Ann Endocrinol (Paris). 2014;75:40–7.

131. Payne AH, Youngblood GL, Sha L, Burgos-Trinidad M, Hammond SH.

Hormonal regulation of steroidogenic enzyme gene expression in

Leydig cells. J Steroid Biochem Mol Biol. 1992;43(8):895–906.

132. Vasta V, Shimizu-Albergine M, Beavo JA. Modulation of Leydig cell function

by cyclic nucleotide phosphodiesterase 8A. Proc Natl Acad Sci U S

A. 2006;103(52):19925–30.

133. Gharani N, Waterworth DM, Batty S, White D, Gilling-Smith C, Conway

GS, et al. Association of the steroid synthesis gene CYP11a with

polycystic ovary syndrome and hyperandrogenism. Hum Mol Genet.

1997;6(3):397–402.

134. Raz E. The function and regulation of vasa-like genes in germ-cell

development. Genome Biol. 2000;1:1–6.

135. Gustafson EA, Wessel GM. Vasa genes: emerging roles in the germ line

and in multipotent cells. BioEssays. 2010;2:626–37.

136. Hickford DE, Frankenberg S, Pask AJ, Shaw G, Renfree MB. DDX4 (VASA)

is conserved in germ cell development in marsupials and monotremes.

Biol Reprod. 2011;85(4):733–43.

137. Georges A, Auguste A, Bessi.re L, Vanet A, Todeschini AL, Veitia RA.

FOXL2: a central transcription factor of the ovary. J Mol Endocrinol.

2013;52:17–33.

138. Schmidt D, Ovitt CE, Anlag K, Fehsenfeld S, Gredsted L, Treier AC, et al.

The murine winged-helix transcription factor Foxl2 is required for

granulosa cell differentiation and ovary maintenance. Development.

2004;131(4):933–42.

139. Georges A, L’H.te D, Todeschini AL, Auguste A, Legois B, Zider A, et al.

The transcription factor FOXL2 mobilizes estrogen signaling to maintain

the identity of ovarian granulosa cells. Elife. 2014;3(November):1–19.
